# 2-(4-Meth­oxy­phenyl)-2-oxoethan­aminium chloride

**DOI:** 10.1107/S1600536812039645

**Published:** 2012-09-22

**Authors:** Hoong-Kun Fun, Wan-Sin Loh, S. Viveka, Dinesha, G. K. Nagaraja

**Affiliations:** a X-ray Crystallography Unit, School of Physics, Universiti Sains Malaysia, 11800 USM, Penang, Malaysia; b Department of Pharmaceutical Chemistry, College of Pharmacy, King Saud University, PO Box 2457, Riyadh 11451, Saudi Arabia; c Department of Chemistry, Mangalore University, Karnataka, India

## Abstract

In the cation of the title compound, C_9_H_12_NO_2_^+^·Cl^−^, the dihedral angle between the 2-oxoethanaminium N—C—C(=O)– plane [maximum deviation = 0.0148 (12) Å] and the benzene ring is 7.98 (8)°. The meth­oxy group is approximately in-plane with the benzene ring, with a C—O—C—C torsion angle of −2.91 (18)°. In the crystal, the cations and chloride anions are connected by N—H⋯Cl and C—H⋯Cl hydrogen bonds, forming a layer parallel to the *bc* plane. A C—H⋯π inter­action further links the layers.

## Related literature

For syntheses and applications of nitro­gen-containing heterocyclic compounds, see: Alvarez-Builla *et al.* (2011 ▶); Katritzky *et al.* (2010 ▶); Chen *et al.* (2011 ▶). For a related structure, see: Zhang *et al.* (2009 ▶). For the stability of the temperature controller used for the data collection, see: Cosier & Glazer (1986 ▶).
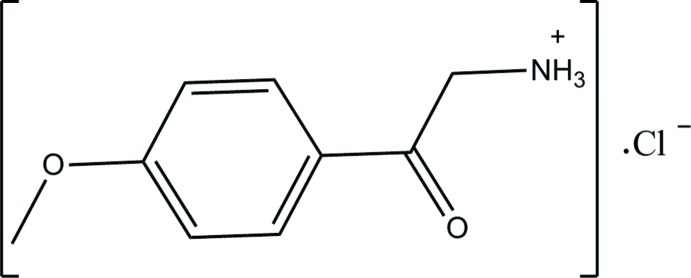


## Experimental

### 

#### Crystal data

C_9_H_12_NO_2_^+^·Cl^−^
*M* *_r_* = 201.65
Monoclinic, 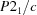

*a* = 12.2822 (8) Å
*b* = 7.1605 (4) Å
*c* = 11.1226 (7) Å
β = 92.435 (1)°
*V* = 977.31 (10) Å^3^
*Z* = 4
Mo *K*α radiation
μ = 0.36 mm^−1^
*T* = 100 K
0.40 × 0.24 × 0.17 mm

#### Data collection

Bruker SMART APEXII DUO CCD area-detector diffractometer
Absorption correction: multi-scan (*SADABS*; Bruker, 2009 ▶) *T*_min_ = 0.870, *T*_max_ = 0.942
10764 measured reflections
2871 independent reflections
2622 reflections with *I* > 2σ(*I*)
*R*_int_ = 0.019

#### Refinement

*R*[*F* ^2^ > 2σ(*F* ^2^)] = 0.035
*wR*(*F* ^2^) = 0.099
*S* = 1.08
2871 reflections
132 parameters
H atoms treated by a mixture of independent and constrained refinement
Δρ_max_ = 0.66 e Å^−3^
Δρ_min_ = −0.31 e Å^−3^

### 

Data collection: *APEX2* (Bruker, 2009 ▶); cell refinement: *SAINT* (Bruker, 2009 ▶); data reduction: *SAINT*; program(s) used to solve structure: *SHELXTL* (Sheldrick, 2008 ▶); program(s) used to refine structure: *SHELXTL*; molecular graphics: *SHELXTL*; software used to prepare material for publication: *SHELXTL* and *PLATON* (Spek, 2009 ▶).

## Supplementary Material

Crystal structure: contains datablock(s) global, I. DOI: 10.1107/S1600536812039645/is5194sup1.cif

Structure factors: contains datablock(s) I. DOI: 10.1107/S1600536812039645/is5194Isup2.hkl

Supplementary material file. DOI: 10.1107/S1600536812039645/is5194Isup3.cml

Additional supplementary materials:  crystallographic information; 3D view; checkCIF report

## Figures and Tables

**Table 1:** Hydrogen-bond geometry (Å, °) *Cg*1 is the centroid of the C2–C7 ring.

*D*—H⋯*A*	*D*—H	H⋯*A*	*D*⋯*A*	*D*—H⋯*A*
N1—H2N1⋯Cl1^i^	0.95 (2)	2.26 (2)	3.2061 (14)	173.6 (15)
N1—H3N1⋯Cl1	0.99 (2)	2.19 (2)	3.1496 (12)	162.6 (19)
N1—H1N1⋯Cl1^ii^	0.97 (2)	2.27 (2)	3.2240 (12)	168.4 (19)
C9—H9*B*⋯Cl1^iii^	0.99	2.69	3.6135 (13)	156
C3—H3*A*⋯*Cg*1^iv^	0.95	2.53	3.3909 (14)	150

Symmetry codes: (i) 
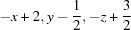
; (ii) 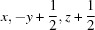; (iii) 
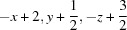
; (iv) 
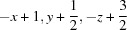
.

**Fig. 1.**
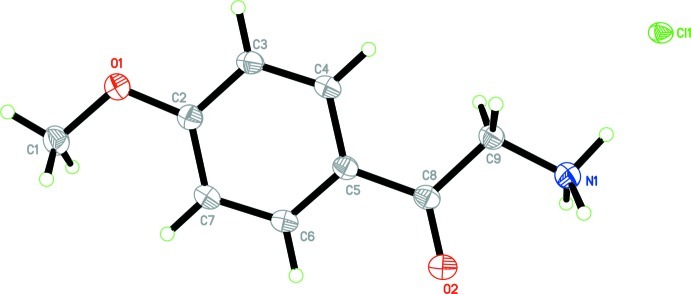
The molecular structure of the title compound, showing 50% probability displacement ellipsoids.

**Fig. 2.**
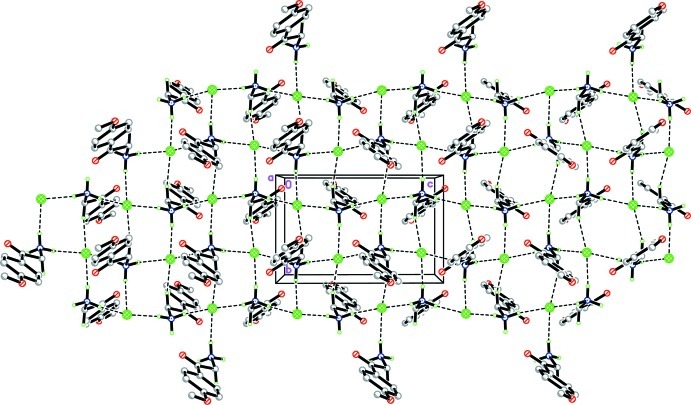
The crystal packing of the title compound, viewed along the a axis, showing the layer parallel to the bc plane. H atoms not involved in the intermolecular interactions (dashed lines) have been omitted for clarity.

## supplementary crystallographic information

### Comment

The synthesis of nitrogen-containing heterocycles has long been a topic of intense research (Alvarez-Builla *et al.*, 2011; Katritzky *et al.*, 2010). This is due, in large part, to the importance of these compounds as drug candidates. The vast majority of new molecular entities (NMEs) contain at least one nitrogen atom in the chemical structure. A subcategory of these compounds are imidazoles, which are notable pharmacophores in a number of areas of discovery chemistry research (Chen *et al.*, 2011). Appropriately, numerous synthetic approaches to these compounds have been published in the literature (Alvarez-Builla *et al.*, 2011). Phenacyl amines are the key intermediate in the synthesis of various ketoamides and also provides a robust synthetic route toward 1*H*-4-substituted imidazole developed using phenacyl amines. Herein we report the synthesis and crystal structure of 2-(4-methoxyphenyl)-2-oxoethanaminium chloride.

The asymmetric unit of the title compound as shown in Fig. 1 consists of one 2-(4-methoxyphenyl)-2-oxoethanaminium cation and one chloride anion. One proton is transferred from the hydrochloric acid to the N atom. The ketone side chain and the methoxy group are coplanar with the benzene ring (C2–C7) with the torsion angles of C6—C5—C8—C9 = 173.82 (11)° and C1—O1—C2—C7 = -2.91 (18)°, respectively. The bond lengths and angles are similar to a related structure (Zhang *et al.*, 2009).

The crystal structure (Fig. 2) is mainly stabilized by N—H···Cl and C—H···Cl hydrogen bonds (Table 1). In the crystal structure, the amine N atom acts as donor whereas the chloride anion acts as acceptor, linking them into a layer parallel to the *bc* plane. A C—H···π interaction (Table 1), involving the benzene ring, further consolidates the crystal structure.

### Experimental

A 40 ml ethanolic solution of 5 mmol 4-methoxy phenacyl bromide was stirred with 5 mmol of HMTA for 10 h. The solid precipitated was filtered and the precipitate was dissolved in HCl and evaporated to dryness to get the crystals. *M.p.*: 433 K.

### Refinement

N-bound H atoms were located in a difference Fourier map and were refined freely [N—H = 0.95 (2) to 0.98 (2) Å]. The remaining H atoms were positioned geometrically (C—H = 0.95 to 0.99 Å) and refined with a riding model with *U*_iso_(H) = 1.2 or 1.5 *U*_eq_(C). A rotating group model was applied to the methyl group. In the final refinement, one outliner, 1 0 0, was omitted.

### Crystal data

**Table:**

C_9_H_12_NO_2_^+^·Cl^−^	*F*(000) = 424
*M**_r_* = 201.65	*D*_x_ = 1.370 Mg m^−^^3^
Monoclinic, *P*2_1_/*c*	Mo *K*α radiation, λ = 0.71073 Å
Hall symbol: -P 2ybc	Cell parameters from 6587 reflections
*a* = 12.2822 (8) Å	θ = 3.3–30.1°
*b* = 7.1605 (4) Å	µ = 0.36 mm^−^^1^
*c* = 11.1226 (7) Å	*T* = 100 K
β = 92.435 (1)°	Block, yellow
*V* = 977.31 (10) Å^3^	0.40 × 0.24 × 0.17 mm
*Z* = 4	

### Data collection

**Table:**

Bruker SMART APEXII DUO CCD area-detector diffractometer	2871 independent reflections
Radiation source: fine-focus sealed tube	2622 reflections with *I* > 2σ(*I*)
Graphite monochromator	*R*_int_ = 0.019
φ and ω scans	θ_max_ = 30.2°, θ_min_ = 3.3°
Absorption correction: multi-scan (*SADABS*; Bruker, 2009)	*h* = −17→17
*T*_min_ = 0.870, *T*_max_ = 0.942	*k* = −10→10
10764 measured reflections	*l* = −15→15

### Refinement

**Table:**

Refinement on *F*^2^	Secondary atom site location: difference Fourier map
Least-squares matrix: full	Hydrogen site location: inferred from neighbouring sites
*R*[*F*^2^ > 2σ(*F*^2^)] = 0.035	H atoms treated by a mixture of independent and constrained refinement
*wR*(*F*^2^) = 0.099	*w* = 1/[σ^2^(*F*_o_^2^) + (0.0509*P*)^2^ + 0.5579*P*] where *P* = (*F*_o_^2^ + 2*F*_c_^2^)/3
*S* = 1.08	(Δ/σ)_max_ = 0.001
2871 reflections	Δρ_max_ = 0.66 e Å^−^^3^
132 parameters	Δρ_min_ = −0.31 e Å^−^^3^
0 restraints	Extinction correction: *SHELXTL* (Sheldrick, 2008), Fc^*^=kFc[1+0.001xFc^2^λ^3^/sin(2θ)]^-1/4^
Primary atom site location: structure-invariant direct methods	Extinction coefficient: 0.027 (3)

### Special details

**Table:**

Experimental. The crystal was placed in the cold stream of an Oxford Cryosystems Cobra open-flow nitrogen cryostat (Cosier & Glazer, 1986) operating at 100.0 (1) K.
Geometry. All e.s.d.'s (except the e.s.d. in the dihedral angle between two l.s. planes) are estimated using the full covariance matrix. The cell e.s.d.'s are taken into account individually in the estimation of e.s.d.'s in distances, angles and torsion angles; correlations between e.s.d.'s in cell parameters are only used when they are defined by crystal symmetry. An approximate (isotropic) treatment of cell e.s.d.'s is used for estimating e.s.d.'s involving l.s. planes.
Refinement. Refinement of *F*^2^ against ALL reflections. The weighted *R*-factor *wR* and goodness of fit *S* are based on *F*^2^, conventional *R*-factors *R* are based on *F*, with *F* set to zero for negative *F*^2^. The threshold expression of *F*^2^ > σ(*F*^2^) is used only for calculating *R*-factors(gt) *etc*. and is not relevant to the choice of reflections for refinement. *R*-factors based on *F*^2^ are statistically about twice as large as those based on *F*, and *R*- factors based on ALL data will be even larger.

### Fractional atomic coordinates and isotropic or equivalent isotropic displacement parameters (Å^2^)

**Table:**

	*x*	*y*	*z*	*U*_iso_*/*U*_eq_	
O1	0.31506 (8)	0.43288 (14)	0.83094 (9)	0.0240 (2)	
O2	0.77620 (8)	0.12046 (16)	1.02231 (9)	0.0271 (2)	
N1	0.93861 (9)	0.16996 (19)	0.87460 (10)	0.0224 (2)	
C1	0.22952 (11)	0.3669 (2)	0.90447 (13)	0.0252 (3)	
H1A	0.1601	0.4227	0.8767	0.038*	
H1B	0.2457	0.4023	0.9884	0.038*	
H1C	0.2245	0.2306	0.8984	0.038*	
C2	0.41825 (10)	0.37782 (18)	0.86212 (11)	0.0187 (2)	
C3	0.49898 (10)	0.43820 (18)	0.78563 (11)	0.0198 (2)	
H3A	0.4795	0.5137	0.7179	0.024*	
C4	0.60660 (10)	0.38863 (18)	0.80810 (11)	0.0191 (2)	
H4A	0.6607	0.4297	0.7555	0.023*	
C5	0.63640 (10)	0.27779 (17)	0.90828 (11)	0.0176 (2)	
C6	0.55558 (10)	0.22213 (17)	0.98526 (11)	0.0186 (2)	
H6A	0.5754	0.1504	1.0546	0.022*	
C7	0.44689 (11)	0.26912 (17)	0.96292 (11)	0.0192 (2)	
H7A	0.3928	0.2280	1.0154	0.023*	
C8	0.74917 (10)	0.21092 (17)	0.93292 (11)	0.0189 (2)	
C9	0.83234 (11)	0.25722 (18)	0.84067 (11)	0.0202 (2)	
H9A	0.8064	0.2111	0.7606	0.024*	
H9B	0.8412	0.3944	0.8355	0.024*	
Cl1	1.05551 (2)	0.22239 (4)	0.63026 (2)	0.01705 (10)	
H1N1	0.9649 (18)	0.215 (3)	0.952 (2)	0.041 (6)*	
H2N1	0.9365 (14)	0.037 (3)	0.8780 (16)	0.029 (5)*	
H3N1	0.9882 (18)	0.196 (3)	0.810 (2)	0.040 (6)*	

### Atomic displacement parameters (Å^2^)

**Table:**

	*U*^11^	*U*^22^	*U*^33^	*U*^12^	*U*^13^	*U*^23^
O1	0.0205 (4)	0.0274 (5)	0.0244 (4)	0.0012 (4)	0.0030 (3)	0.0044 (4)
O2	0.0252 (5)	0.0328 (5)	0.0232 (5)	0.0012 (4)	0.0017 (4)	0.0083 (4)
N1	0.0209 (5)	0.0255 (6)	0.0210 (5)	0.0000 (4)	0.0023 (4)	0.0003 (4)
C1	0.0207 (6)	0.0270 (7)	0.0281 (6)	−0.0017 (5)	0.0041 (5)	0.0015 (5)
C2	0.0211 (5)	0.0171 (5)	0.0180 (5)	0.0001 (4)	0.0018 (4)	−0.0018 (4)
C3	0.0242 (6)	0.0186 (6)	0.0166 (5)	0.0008 (4)	0.0026 (4)	0.0019 (4)
C4	0.0231 (6)	0.0184 (5)	0.0161 (5)	−0.0016 (4)	0.0040 (4)	0.0010 (4)
C5	0.0200 (5)	0.0171 (5)	0.0157 (5)	−0.0016 (4)	0.0015 (4)	−0.0010 (4)
C6	0.0235 (6)	0.0175 (5)	0.0150 (5)	−0.0013 (4)	0.0018 (4)	0.0004 (4)
C7	0.0223 (6)	0.0189 (6)	0.0168 (5)	−0.0021 (4)	0.0043 (4)	−0.0002 (4)
C8	0.0215 (5)	0.0178 (5)	0.0176 (5)	−0.0022 (4)	0.0018 (4)	−0.0005 (4)
C9	0.0209 (6)	0.0209 (6)	0.0189 (5)	−0.0001 (4)	0.0028 (4)	0.0010 (4)
Cl1	0.02011 (15)	0.01794 (16)	0.01333 (15)	0.00302 (9)	0.00337 (9)	0.00102 (9)

### Geometric parameters (Å, º)

**Table:**

O1—C2	1.3582 (15)	C3—C4	1.3812 (17)
O1—C1	1.4384 (16)	C3—H3A	0.9500
O2—C8	1.2208 (16)	C4—C5	1.4039 (17)
N1—C9	1.4814 (17)	C4—H4A	0.9500
N1—H1N1	0.96 (2)	C5—C6	1.3962 (17)
N1—H2N1	0.95 (2)	C5—C8	1.4800 (17)
N1—H3N1	0.98 (2)	C6—C7	1.3888 (18)
C1—H1A	0.9800	C6—H6A	0.9500
C1—H1B	0.9800	C7—H7A	0.9500
C1—H1C	0.9800	C8—C9	1.5150 (18)
C2—C7	1.3975 (17)	C9—H9A	0.9900
C2—C3	1.4020 (17)	C9—H9B	0.9900
			
C2—O1—C1	117.11 (10)	C3—C4—H4A	119.9
C9—N1—H1N1	110.3 (13)	C5—C4—H4A	119.9
C9—N1—H2N1	114.0 (11)	C6—C5—C4	118.68 (11)
H1N1—N1—H2N1	107.7 (16)	C6—C5—C8	118.57 (11)
C9—N1—H3N1	107.4 (13)	C4—C5—C8	122.70 (11)
H1N1—N1—H3N1	113.7 (19)	C7—C6—C5	121.61 (11)
H2N1—N1—H3N1	103.8 (17)	C7—C6—H6A	119.2
O1—C1—H1A	109.5	C5—C6—H6A	119.2
O1—C1—H1B	109.5	C6—C7—C2	119.06 (11)
H1A—C1—H1B	109.5	C6—C7—H7A	120.5
O1—C1—H1C	109.5	C2—C7—H7A	120.5
H1A—C1—H1C	109.5	O2—C8—C5	122.85 (11)
H1B—C1—H1C	109.5	O2—C8—C9	119.99 (12)
O1—C2—C7	124.53 (11)	C5—C8—C9	117.16 (10)
O1—C2—C3	115.59 (11)	N1—C9—C8	110.32 (10)
C7—C2—C3	119.88 (12)	N1—C9—H9A	109.6
C4—C3—C2	120.47 (11)	C8—C9—H9A	109.6
C4—C3—H3A	119.8	N1—C9—H9B	109.6
C2—C3—H3A	119.8	C8—C9—H9B	109.6
C3—C4—C5	120.27 (11)	H9A—C9—H9B	108.1
			
C1—O1—C2—C7	−2.91 (18)	C5—C6—C7—C2	−1.25 (19)
C1—O1—C2—C3	177.42 (11)	O1—C2—C7—C6	−179.90 (12)
O1—C2—C3—C4	−179.30 (11)	C3—C2—C7—C6	−0.23 (18)
C7—C2—C3—C4	1.01 (19)	C6—C5—C8—O2	−5.45 (19)
C2—C3—C4—C5	−0.31 (19)	C4—C5—C8—O2	177.16 (12)
C3—C4—C5—C6	−1.13 (18)	C6—C5—C8—C9	173.82 (11)
C3—C4—C5—C8	176.25 (11)	C4—C5—C8—C9	−3.57 (17)
C4—C5—C6—C7	1.93 (19)	O2—C8—C9—N1	3.06 (17)
C8—C5—C6—C7	−175.56 (11)	C5—C8—C9—N1	−176.23 (11)

### Hydrogen-bond geometry (Å, º)

*Cg*1 is the centroid of the C2–C7 ring.

**Table:**

*D*—H···*A*	*D*—H	H···*A*	*D*···*A*	*D*—H···*A*
N1—H2*N*1···Cl1^i^	0.95 (2)	2.26 (2)	3.2061 (14)	173.6 (15)
N1—H3*N*1···Cl1	0.99 (2)	2.19 (2)	3.1496 (12)	162.6 (19)
N1—H1*N*1···Cl1^ii^	0.97 (2)	2.27 (2)	3.2240 (12)	168.4 (19)
C9—H9*B*···Cl1^iii^	0.99	2.69	3.6135 (13)	156
C3—H3*A*···*Cg*1^iv^	0.95	2.53	3.3909 (14)	150

Symmetry codes: (i) −*x*+2, *y*−1/2, −*z*+3/2; (ii) *x*, −*y*+1/2, *z*+1/2; (iii) −*x*+2, *y*+1/2, −*z*+3/2; (iv) −*x*+1, *y*+1/2, −*z*+3/2.
